# Data-Driven Analysis for Safe Ship Operation in Ports Using Quantile Regression Based on Generalized Additive Models and Deep Neural Network

**DOI:** 10.3390/s21248254

**Published:** 2021-12-10

**Authors:** Hyeong-Tak Lee, Hyun Yang, Ik-Soon Cho

**Affiliations:** 1 Ocean Science and Technology School, Korea Maritime and Ocean University, Busan 49112, Korea; gudxkr518@kmou.ac.kr; 2 Korea Ocean Satellite Center, Korea Institute of Ocean Science & Technology, Busan 49112, Korea; yanghyun@kiost.ac.kr; 3 Division of Navigation Convergence Studies, Korea Maritime and Ocean University, Busan 49112, Korea

**Keywords:** automatic information system, deep learning, deep neural network, generalized additive models, quantile regression, safe ship operation, ship trajectories

## Abstract

Marine accidents in ports can cause loss of human life and property and have negative material and environmental impacts. In South Korea, due to a pier collision accident of a large container ship in Busan New Port of South Korea, the need for safe ship operation guidelines in ports emerged. Therefore, to support quantitative safe ship operation guidelines, ship trajectory data based on automatic information system information have been used. However, because this trajectory information is variable and uncertain due to various situations arising during a ship’s navigation, there is a limit to deriving results through traditional regression analysis. Considering the characteristics of these data, we analyzed ship trajectories through quantile regression using two models based on generalized additive models and neural networks corresponding to deep learning. Among the automatic information system information, the speed over ground, course over ground, and ship’s position were analyzed, and the model was evaluated based on quantile loss. Based on this study, it is possible to suggest safe operation guidelines for the position, speed, and course of the ship. In addition, the results of this work can be further developed as a manual for the in-port-autonomous operation of ships in the future.

## 1. Introduction

Maritime accidents in ports not only cause loss of human life and physical damage to ships, but also have economic consequences for maritime transportation and environmental consequences in ports [1]. As a result of the introduction of increasingly massive ships, greater safety measures are required in the operation of ships in ports [2]. In recent years, maritime accidents resulting from human factors in the piloting process, such as the inability to control excessive speed of the ship in a timely manner or the inability to secure a sufficiently safe distance from the pier, have become frequent [3]. A typical example is the pier collision accident of a 13,900 TEU container ship that was entering Busan New Port in April 2020 [4]. In the accident, a gantry crane was completely destroyed and three were partially damaged. Considerable other damages and injuries to cargo workers were also incurred.

A special investigation report about this accident was prepared by the Korea Maritime Safety Tribunal (KMST) of the Ministry of Oceans and Fisheries in the Republic of Korea [3]. The role of a pilot with professional knowledge about a port is extremely important when a ship enters the port [5]. Accordingly, the report presented a proposal to prepare a standard pilot manual to minimize the possibility of accidents caused by human factors, such as the skill gap between pilots or differences in ship-handling methods. In particular, it is necessary to prepare safety procedures for ship operation, such as safe navigation in dangerous sections and safe velocity for pier approach, based on many piloting cases in the same port.

Ship trajectory data are required to collect information about various cases of piloting ships in a port. Trajectory data have become important for big-data analysis due to advances in science and technology, and are widely used for purposes such as target tracking, behavior analysis, and navigation [6]. Ship trajectory data can be collected using an automatic identification system (AIS). AIS data include ship navigation status information such as the position, speed over ground (SOG), and course over ground (COG) of the ship [7].

AIS ship trajectory data are a crucial data source in research on the safe operation of ships. Lee et al. extracted the vessel traffic route through quantitative analysis based on AIS data for the design of a safe route considering the ship characteristics [8]. Son et al. analyzed the range of the safe distance for ships sailing under a bridge across a waterway through AIS-based tracking [9]. Huang et al. analyzed the crossing-line and used Monte Carlo methods based on AIS data for navigation safety assessment for an approaching channel [10]. Active research is ongoing on the use of artificial intelligence techniques for ship trajectories based on big AIS data. Namgung and Kim predicted a ship’s trajectory according to future tidal conditions using support vector regression to reduce maritime accidents [11]. Deep-learning-based ship trajectory prediction methods, such as long short-term memory (LSTM), for maritime navigation early warning and safety are being studied [12,13]. In some studies, clustering algorithms, such as density-based spatial clustering of applications with noise (DBSCAN), have been used to analyze the patterns of ship trajectories [14,15]. Lee et al. analyzed the patterns of the trajectories of ships entering and leaving Busan New Port using DBSCAN; their study was significant for analyzing the patterns of trajectories of ships in the port rather than in the ocean [16].

However, few studies have proposed quantitative analyses using ship trajectory data for safe operation in port. To analyze the safety procedures for ship operation, data such as the position, speed, and course of the ship, which are information included in the AIS, should be used [17]. Therefore, in this study, the ship’s position, SOG, and COG data were regression analyzed to suggest appropriate guidelines for safe ship operation.

Ship operation in a port is affected by the size of the ship, the amount of traffic, the pilot’s tendency, and environmental conditions [5], [18], thereby resulting in variable and uncertain ship trajectory data. If only the central tendency is considered when quantifying the datasets with such variability and uncertainty, the datasets cannot be properly represented. Therefore, the traditional regression analysis method that provides information on the effect of the independent variable on the mean value of the dependent variable has limitations with regard to framing guidelines for safe ship operation. Because of these limitations, quantile regression, which provides a regression model for conditional quantiles of the dependent variable, was used in this study. Quantile regression analysis focuses on the entire distribution by estimating the effect on the entire distribution of the response variable, instead of on the mean of the independent variable [19]. Because ship maneuvering is influenced by several factors, such as weather and traffic flow, proposing maneuvering guidelines based on an average of data is inappropriate. Therefore, in this study, quantile regression was applied to ship trajectory data, and the operating range of ships according to quantiles was determined to serve as a maneuvering guideline. Dinparast Djadid et al. used Bayesian quantile regression to model a driver’s response time to reclaim control in automation [19]. Zou et al. used quantile regression to investigate factors influencing the time taken to clear road traffic incidents and reported that it can be used to make inferences about the effect of explanatory variables on different quantiles of the incidents’ duration distribution [20].

The application of quantile regression in various means in combination with generalized additive models (GAMs) and deep learning is being explored. Murphy et al. analyzed water quality using GAMs combined with quantile regression [21]. Dinga et al. proposed an analysis method using quantile GAMs [22]. Quantile regression neural networks (QRNNs), which are based on deep learning and artificial intelligence techniques, are also being used in many studies. Further, with regard to load forecasting, research on analyzing the volatility and uncertainty of load data using QRNNs is ongoing [23,24], and in other fields, QRNNs have been applied to a breast cancer dataset [25].

The goal of this study was to provide guidelines for safe ship operation in ports by applying quantile regression using AIS-based ship trajectory data to resolve the volatility and uncertainty in ship maneuvering. Quantile regression in the analysis was utilized by combining GAMs and neural networks. Accordingly, we propose a quantitative standard guideline for safe ship operation in ports to minimize the possibility of ship accidents caused by human factors.

## 2. Materials and Methods

**Figure 1:**
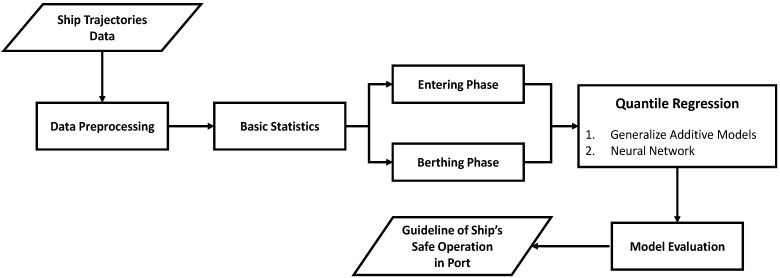
Flowchart of the study.

Figure 1 shows a flowchart of this study. First, AIS-based ship-arriving trajectories were collected at a target pier. The collected data were preprocessed to make the data suitable for analysis. Subsequently, the data characteristics were identified and visualized using basic data statistics. In the next step, the data were classified and modeled as the entering phase and berthing phase data according to the characteristics of the target pier. The technique utilizes GAMs and deep neural networks based on quantile regression. Further, a suitable model was selected through model evaluation and, finally, guidelines for safe ship operation in the port were proposed.

### 2.1. Ship Trajectory Data

**Figure 2:**
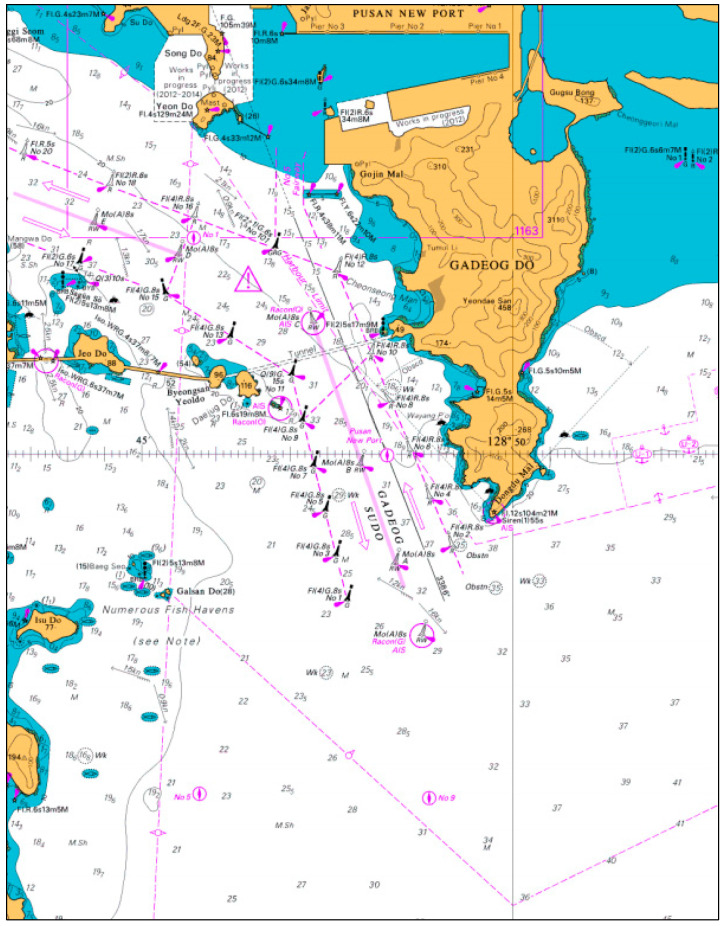
Geographical characteristics of Busan New Port: British Admiralty Chart.

We analyzed the data for Busan New Port in the Republic of Korea. The Busan New Port is a container ship port at which the aforementioned crane collision accident had occurred. The British Admiralty Chart of Busan New Port is shown in Figure 2.

Ship trajectory data were collected based on the AIS information of ships arriving at this port. In the ‘Navigation Rules of Busan Port (Notice of Busan Regional Office of Oceans and Fisheries)’, only the ship’s speed, such as the passing speed for each section and the moving speed within the port, is presented as a procedure. However, to propose a ship maneuvering guideline in port, the route and ship’s position under operation, including speed, should be given [16]. This port is relatively protected against the effects of weather compared to other ports, and the port is closed in the event of severe weather and poor visibility. Yoon et al. found that berthing of large containers at Busan New Port does not pose a significant risk, except in the event of severe weather [26]. Therefore, the variables considered in this study were the time and date, ship’s position, SOG, and COG obtained from the AIS information. The data collection period was fixed considering the time of the gantry crane collision. Data were collected for four months from January 2020. The target ship type considered in the study was large container ships with a gross tonnage of 100k or more, similar to the ship involved in the accident. To consider all cases in which the ship was safely berthing, the weather conditions for the period were not separated. The details of the collected data are summarized in Table 1.

### 2.2. Data Preprocessing and Statistics

In terms of data mining, data preprocessing is an essential step for improving the performance of analysis models [27]. As AIS data have information errors and reception errors due to receiving sensitivity about ships, preprocessing through data deletion is essential [28]. In addition, AIS data are characterized by different data reception intervals depending on the speed and changing course of the ship [29]. For example, the dynamic information of Class-A AIS used by SOLAS (International Convention for the Safety of Life at Sea) ships is received at intervals of 10 s for ships sailing under 14 knots, and at intervals of 3.3 s when the ship changes its course. In addition, because the arrival time of each ship is different, it is necessary to unify the unit used for the change in position with time when analyzing ship trajectories. Therefore, in this study, the data-cleaning and data-scaling methods were applied for data preprocessing.

Data cleaning is a preprocessing method that deletes noisy data and missing values to make the data suitable for analysis [27]. Missing values caused by AIS errors were removed using the list-wise delete method [30]. A ship entering Busan New Port was preprocessed with the AIS data from the position corresponding to maneuvering from the pilot station to the berthing. Data before the pilot station do not have regularities such as drifting and anchoring, and the port entry of the ship starts when the pilot is on board. Hence, the data cleansing was performed for latitude 34.93°N.

Data scaling is the process of standardizing data units. When analyzing data, the data should be normalized to avoid any error in the analysis results due to differences in units [27]. In this study, min–max normalization was used as the data-scaling methods. When the information of each ship is defined as S(x), the time corresponding to the starting point of the section is defined as $S\left(x_{min}\right)$ and the last point as $S\left(x_{max}\right)$, and the normalization proceeds as shown in Equation (1):

(1) $$MinMax\;normalization=\frac{S\left(x\right)-S\left(x_{min}\right)}{S\left(x_{max}\right)-S\left(x_{min}\right)}$$

The number of vessel arrivals in the ship trajectory dataset was 50 in this study. For the middle of the berth at the target pier of Busan New Port at the time, a ship can dock at the pier after passing a small island called Todo. When a ship passes Todo, it is classified as a case of berthing by passing to the left or a case of berthing by passing to the right. The frequency results are listed in Table 2.

The purpose of this study was to propose a maneuvering guideline by analyzing the ranges according to quantiles for SOG, COG, and ship’s position in the entire ship trajectory data. Therefore, cross-validation such as dividing into training and testing datasets was not conducted.

### 2.3. Quantile Regression

Multiple linear regression is a basic and standard approach that uses the values of multiple variables to describe or predict the mean value of a scale outcome. In contrast, quantile regression models the relationship between a set of independent variables and a specific percentile (or “quantile”) of a dependent variable; that is, quantile regression is a linear model for the conditional τ quantile of the dependent variable, unlike the traditional regression analysis method that provides information on the influence of the independent variable on the mean value of the dependent variable [31]. This regression model is closely related to the model for the conditional median, and it is possible to estimate the conditional median of predictions and data by minimizing the mean absolute error [32]. The conditional quantiles of the distribution can be obtained by applying an asymmetric weight using the tilted absolute value function.

The tilted absolute value function is also called the pinball loss function and is as shown in Equation (2):

(2) $$\rho_{\tau }\left(u\right)=\{\begin{array}{c} \tau u\\ \left(\tau -1\right)u \end{array}\;\begin{array}{c} if\;u\geq 0\\ if\;u<0 \end{array}$$

where 0<τ<1. Assuming that the analysis variable is $x_{i}\left(t\right)\;\left(i=1,\ldots ,I\right)$, the slope is $m_{i}$, and the intercept is b, the linear regression analysis formula for the τ quantile ${\hat{y}}_{t}$ is as shown in Equation (3):

(3) $${\hat{y}}_{\tau }=\overset{I}{\underset{i=1}{\sum }}m_{i}x_{i}\left(t\right)+b$$

Here, when the observation value at time t is defined as y(t) (t=1,…, N), the equation for estimating the quantile regression error function by minimizing the quantile loss function is as shown in Equation (4):

(4) $$E_{\tau }=\frac{1}{N}\overset{N}{\underset{t=1}{\sum }}\rho_{\tau }(y\left(t\right)-{\hat{y}}_{\tau }\left(t\right))$$

In general, quantile regression is applied to a continuous variable and applied to a linear model, but it can also be applied when the parameter is nonlinear [33]. Such quantile regression can be used to obtain the confidence interval for the analysis result of the model. This regression is suitable for this study because it is less sensitive to outliers [34].

### 2.4. Genealized Additive Models

A GAM is a statistical model that mixed the properties of the generalized linear model with the additive model, and is a linear model that allows nonlinear functions of each variable using a smoothing function [35]. GAMs relax the constraint that the relationship must be a simple weighted sum, and instead assumes that the result can be modeled as a sum of arbitrary features of each feature. GAMs are generally suitable for establishing relationships between complex types of data that cannot be easily represented by linear or nonlinear models or for analyzing models without any special conditions. Therefore, it is appropriate to use GAMs because the ship trajectory data used in this study have uncertainty and volatility.

The general formula for multiple linear regression models is shown in Equation (5):

(5) $$y_{i}=\beta_{0}+\beta_{1}\chi_{i1}+\beta_{2}\chi_{i2}+\cdots +\beta_{p}\chi_{ip}+\epsilon_{i}$$

To consider the nonlinear relationship between each explanatory variable and the response variable, we replace $\beta_{j}\chi_{ij}$ with the smooth nonlinear function $f_{j}\left(x_{ij}\right)$ in the multiple linear regression model to obtain Equation (6):

(6) $$y_{i}=\beta_{0}+f_{1}\left(x_{i1}\right)+f_{1}\left(x_{i1}\right)+\cdots +f_{p}\left(x_{ip}\right)+\epsilon_{i}$$

Equation (6) can be rewritten as Equation (7):

(7) $$y_{i}=\beta_{0}+\overset{p}{\underset{j=1}{\sum }}f_{j}\left(x_{ij}\right)+\epsilon_{i}$$

In this case, $f_{j}$ is an arbitrary function of each explanatory variable $x_{ij}$, and a nonparametric smoothing function is used [36]. Notably, GAMs are data based, not model based, and the fitted values of the results are not derived from an a priori model. Further, as there is no limitation of the shapes available in the parameter class, the data can determine the shape of the response curve. Therefore, this method can provide suitable results for analyzing ship trajectories.

### 2.5. Quantile Regression Newral Network

The artificial neural network is a suitable model for multiple regression problems with nonlinear transformations through complex linkages and activation functions of variables [37]. However, because the existing neural network only provides one output result at a time, it is not possible to derive prediction results according to quantiles [25]. Therefore, a QRNN was proposed. In particular, because NNs can output accurate information based on complex data, it was used to analyze ship trajectory data in this study. The hidden layer of the neural network is dense and connected by nodes. The output of the hidden layer is obtained by applying an activation function between the input value, the weight of the hidden layer, and the bias of the hidden layer [38]. When the input data value is $x_{j}\left(j=1,\;2,\;\ldots ,\;J\right)$, the output of the $k^{th}$ (k=1,2,…,K) node for the first hidden layer is as shown in Equation (8):

(8) $$g_{k}=f_{1}\left(\overset{J}{\underset{j=1}{\sum }}x_{j}w_{jk}^{\left(h\right)}+b_{k}^{\left(h\right)}\right)$$

The output of the $l^{th}$(l=1,2,…,L) node in the second hidden layer is as shown in Equation (9):

(9) $$h_{l}=f_{2}\left(\overset{K}{\underset{k=1}{\sum }}g_{j}w_{kl}^{\left(h\right)}+b_{l}^{\left(h\right)}\right)$$

where $f_{1}$ and $f_{2}$ denote activation functions, and $w^{\left(h\right)}$ and $b^{\left(h\right)}$ denote the weight and bias of the hidden layer, respectively. The output layer of the neural network can be expressed as shown in Equation (10) as a single node with a linear activation function that estimates the $\tau^{th}$ quantile for the $i^{th}$ subject:

(10) $${\hat{Q}}_{i}^{\left(\tau \right)}=\overset{L}{\underset{l=1}{\sum }}h_{l}w_{l}^{\left(o\right)}+b^{\left(o\right)}$$

**Figure 3:**
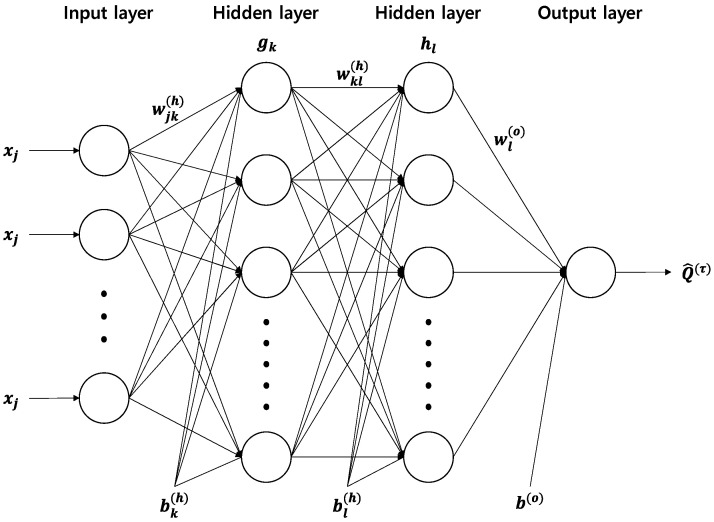
Overview of the quantile regression neural network (QRNN) architecture used in this study.

Here, $w^{\left(o\right)}$ and $b^{\left(o\right)}$ represent the weight and bias of the output layer, respectively. The basic model architecture of the QRNN is shown in Figure 3. In this study, the number of hidden layers and neurons was investigated in each case to determine the best model.

In this study, the Exponential Linear Unit (ELU) was used as the activation function. The ELU can increase the learning speed and classification accuracy of the deep neural network [39]. The ELU outputs the input value without refinement in the positive part to avoid the problem of gradient loss. In the Rectified Linear Unit (ReLU), which is the most widely used among neural network activation functions, the negative part of the function graph is in the form of unsaturation; in contrast, in the ELU, this part is in the form of saturation.

The loss function used in the QRNN model was applied as a quantile loss value, as shown in Equation (4). In addition, the optimizer used the Adaptive Moment Estimation (Adam). Adam has an advantage over previous optimizers because it uses different sizes of updates for each parameter [40].

### 2.6. Model Evaluation

In this study, for the model evaluation of quantile GAMs and the QRNN, the quantile loss was used as the evaluation index [41]. In general regression analysis, if the mean absolute error (MAE), which is a representative index for evaluating the accuracy of the outcome variable, is minimized, the median regression line (0.5-quantile) is obtained. The quantile loss is the weighted MAE calculated to determine the *τ*-quantile. Therefore, this index is suitable for evaluating quantile regression. The quantile loss is defined as shown in Equation (11):

(11) $$Loss\left(\tau \right)=\frac{1}{N}\sum_{i=1}^{N}\rho_{\tau }(y_{i}\left(\tau |x\right)-{\hat{y}}_{i}\left(\tau |x\right))$$

(12) $$MAE\left(\tau \right)=\frac{1}{N}\sum_{i=1}^{N}\left|y_{i}\left(\tau |x\right)-{\hat{y}}_{i}\left(\tau |x\right)\right|$$

where ${\hat{y}}_{i}\left(\tau |x\right)$ is the prediction of the true conditional quantile $y_{i}\left(\tau |x\right)$. Loss(*τ*) depends on asymmetric loss, as shown in Equation (4), and the smaller the measured value of Loss(*τ*), the better the method.

## 3. Experiments and Results

In the experiments, modeling was performed using quantile GAMs and a QRNN with the constructed data. In addition, a suitable model was selected by evaluating the model performances. The experimental platform was a PC terminal, and the programming implementation of the model was completed using Python 3.7.3, pygam 0.8.0, and TensorFlow 1.15.0.

### 3.1. Data Preprocessing and Statistics

The AIS-based ship trajectories data were collected and then preprocessed to make them suitable for application for the quantile regression model. Cleaning was completed based on the pilot station latitude of 34.93° N, and the ship’s position, SOG, and COG data were normalized. The normalization or scaling result was input during the modeling with GAMs and the QRNN. Table 3 summarizes the minimum and maximum values for each datum; values normalized to a value between 0 and 1 were multiplied by 100 for ensuring the clarity of analysis results and expressed as a value between 0 and 100.

**Figure 4:**
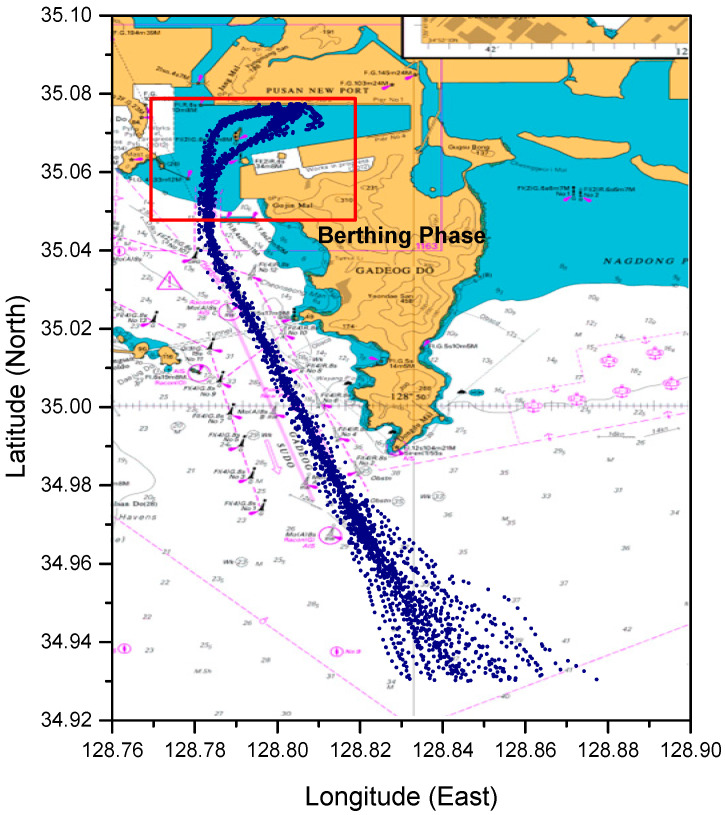
Ship trajectory data plotted on the British Admiralty Chart after completing the preprocessing.

A visualization of the preprocessed dataset is shown in Figure 4. To enter Busan New Port, the ship enters the waterway, Gadeog Sudo, after a pilot experienced in berthing at the port has boarded the ship. The arriving vessel must navigate to the right side of Gadeog Sudo. After the vessel has passed the Gadeog Sudo, it will pass through the breakwater to access the pier. Accordingly, based on the latitude 34.05° N point passing through the east breakwater, the ship trajectories in the dataset were classified into the entering phase and berthing phase. In the berthing phase, the ship employs a tug boat to control the ship’s speed and course. The ships pass by Todo to berth at the target pier. The ship trajectory data used in this study show that ships navigate toward the north according to the passage of time. Therefore, in this study, the SOG, COG, and longitude were analyzed based on changes in the latitude.

**Figure 5:**
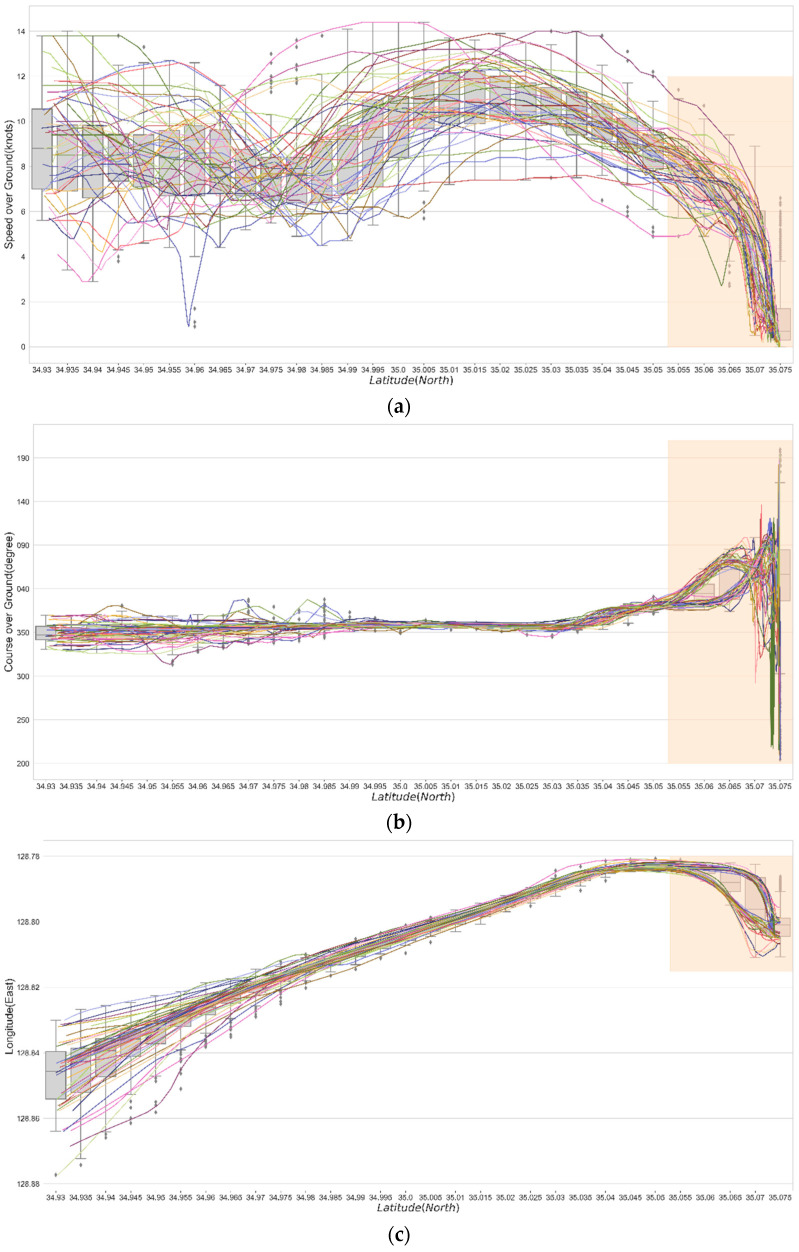
Visualization of ship trajectory data into boxplot and line plot based on latitude: (**a**) speed over ground (SOG); (**b**) course over ground (COG); and (**c**) longitude. The berthing phase part is marked by shading.

A basic statistic was considered to understand the characteristics of AIS information for the entire process of the arrival of a ship. To understand the changes in the SOG, COG, and longitude information according to the latitude, the scatter data of 50 ships were connected with a line and visualized as a line plot, as shown in Figure 5. In addition, from the starting point of ship trajectories, i.e., latitude 34.93° N, the characteristics of the dataset were visualized by a boxplot in 0.05 units. Figure 5a shows the changes in the SOG: the SOG decreases in the latitude range of 34.97° N–34.98° N as the ships enter Gadeog Sudo. Thereafter, the SOG gradually increases as the ships pass through Gadeog Sudo, and then decrease to allow berthing after completing the passage through Gadeog Sudo. Figure 5b shows the changes in COG. The analysis showed that ships generally maneuvered north before the berthing phase. Thereafter, the courses can be classified based on two tendencies when passing by Todo and, just before berthing, a large deviation appears because vessels are precisely maneuvered using a tugboat and an engine. In Figure 5c, which shows the change in longitude, the y-axis is reversed for visual understanding. The descriptive statistics shown by grouping the ship trajectories data into 0.05-units of latitude are summarized in Table 4. Although the characteristics of the dataset can be identified through basic statistics, there is a limit to analyzing ship trajectories with volatility and uncertainty for the purpose of this study. Therefore, the results were derived using quantile GAMs and the QRNN.

### 3.2. Modeling and Evaluation

Before modeling to suggest guidelines for safe ship operation in a port, the dataset was divided into data corresponding to the entering phase and those corresponding to the berthing phase. Depending on the berthing phase, the ships passing by Todo were classified into those that maneuvered to the left and those that maneuvered to the right. This classification was performed to suggest clear and specific guidelines for each section through which the ships enter Busan New Port. In this study, the dependent variable was the latitude, and the independent variables were SOG, COG, and longitude. The ship trajectory data divided into three categories were modeled according to each independent variable.

#### 3.2.1. Modeling of Generalized Additive Models

**Figure 6:**
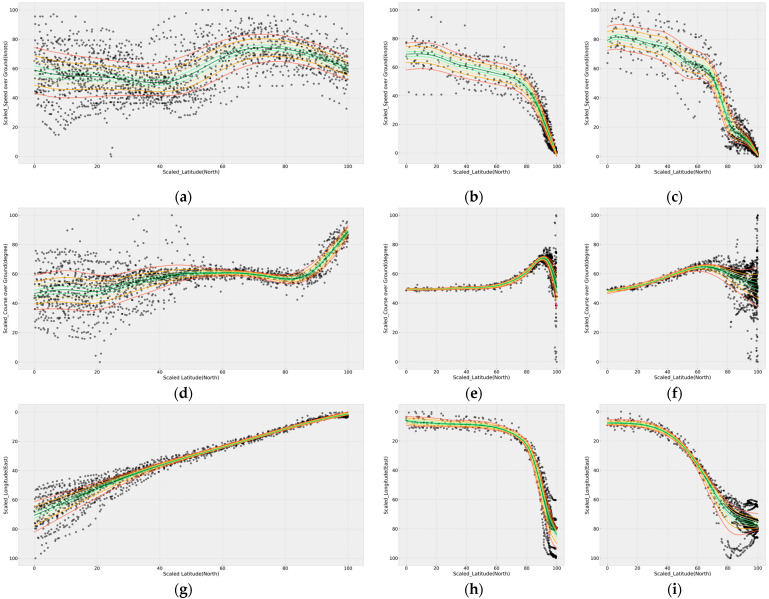
Results of modeling with quantile generalized additive models (GAMs): (**a**,**d**,**g**) SOG, COG, and longitude respectively, in the entering phase; (**b**,**e**,**h**) SOG, COG, and longitude, respectively, when passing Todo to the left in the berthing phase; and (**c**,**f**,**i**) SOG, COG, and longitude, respectively, when passing Todo to the right, in the berthing phase. Furthermore, 0.1 and 0.9 quantiles are indicated by red, 0.2 and 0.8 by orange, 0.3 and 0.7 by yellow, 0.4 and 0.6 by yellow-green, and 0.5 by green lines.

Figure 6 shows the results of quantile regression of the dataset using GAMs. All results of quantile modeling using GAMs were statistically significant (*p*-value < 0.001). Based on the minimum and maximum values listed in Table 3, the x-axis represents the scaled latitude, and the y-axis represents the result of scaling each information. Figure 6a–c shows the results of applying the SOG information to the GAMs by applying the entering phase, the berthing phase of passing by Todo to the left, and the berthing phase of passing by Todo to the right, respectively. Figure 6d–f shows the COG, and Figure 6g–i shows the analysis results for longitude. In particular, GAMs are characterized by smooth lines due to the application of the smoothing function.

#### 3.2.2. Modeling of the Quantile Regression Neural Network

**Figure 7:**
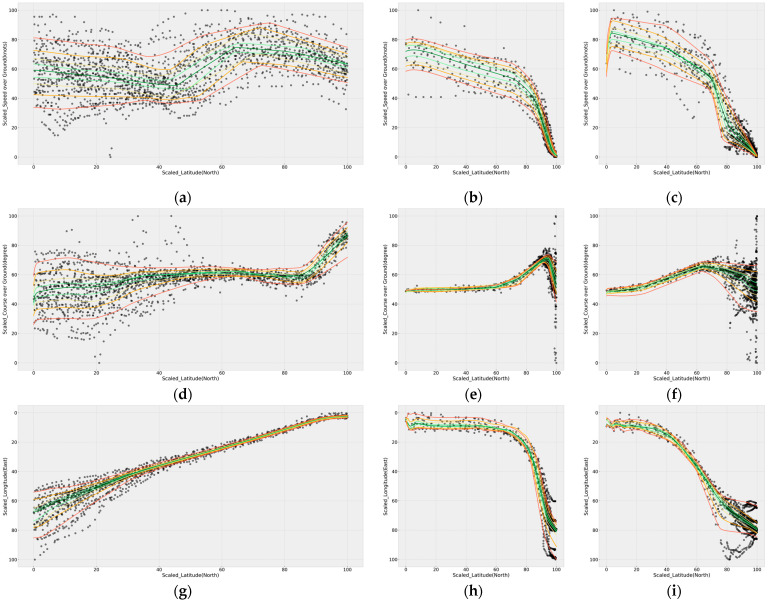
Results of QRNN modeling: (**a**,**d**,**g**) SOG, COG, and longitude, respectively, in the entering phase; (**b**,**e**,**h**) SOG, COG, and longitude, respectively, when passing Todo to the left in the berthing phase; and (**c**,**f**,**i**) SOG, COG, and longitude, respectively, when passing Todo to the right in the berthing phase. Furthermore, 0.1 and 0.9 quantiles are indicated by red, 0.2 and 0.8 by orange, 0.3 and 0.7 by yellow, 0.4 and 0.6 by yellow-green, and 0.5 by green lines.

By comparison, the QRNN is slightly more affected by the distribution of the dataset than GAMs, and hence, the QRNN fitting line is not smooth. The number of hidden layers and the appropriate number of neurons in each hidden layer in the QRNN model determined the most optimal model among 16 cases (Table 5). The QRNN corresponding to 0.5-quantile was modeled for each case using the SOG data from the entering phase, and the optimal model was determined using the MAE. Therefore, according to the results of Table 5, four hidden layers and 16 neurons were determined as the most optimal QRNN modeling. Further, the computational time required was the longest at 15 s, although the analysis was fast and there was minor difference from the other cases. Figure 7 shows the result of visualizing QRNN modeling and shows the analysis results of SOG, COG, and longitude for each phase.

#### 3.2.3. Evaluation

**Figure 8:**
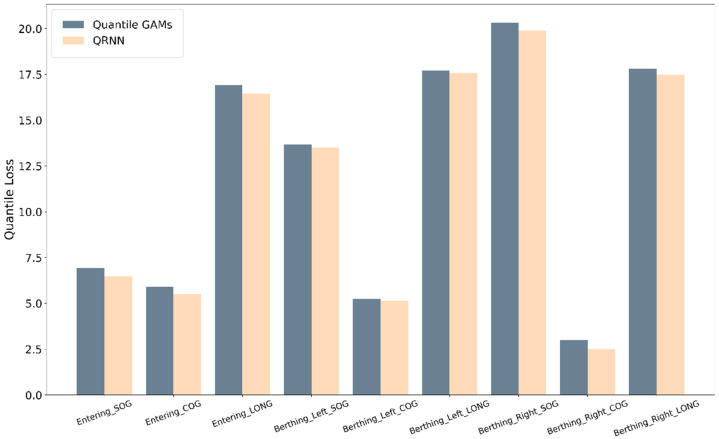
Result of visualizing the comparison of the average values of loss by quantiles as a bar plot: GAMs and QRNN model.

Table 6 lists the performance evaluation results of quantile GAMs and QRNN models. All quantile loss values were derived according to 0.1–0.9 fitting lines of AIS information for each phase. The result of comparing the average value of the loss for each quantile was visualized as a bar plot, as shown in Figure 8. In general, the loss value of QRNN was analyzed to achieve better performance than that of the GAMs. The analysis result indicates that the GAMs and QRNN model is suitable for analysis and is feasible for use as a guideline for safe ship operation based on ship trajectories. However, the QRNN model can yield slightly more accurate results than GAMs.

## 4. Discussion

Ship trajectory data were analyzed using quantile regression-based GAMs and a QRNN for the safe operation of ships in ports. This methodology is meaningful because it proposes the ship maneuvering method in the port as a data-based quantitative numerical value, unlike the traditional system of handing down the information in an apprentice system among pilots. Thus, this methodology presents a new approach to ship trajectory data analysis.

**Figure 9:**
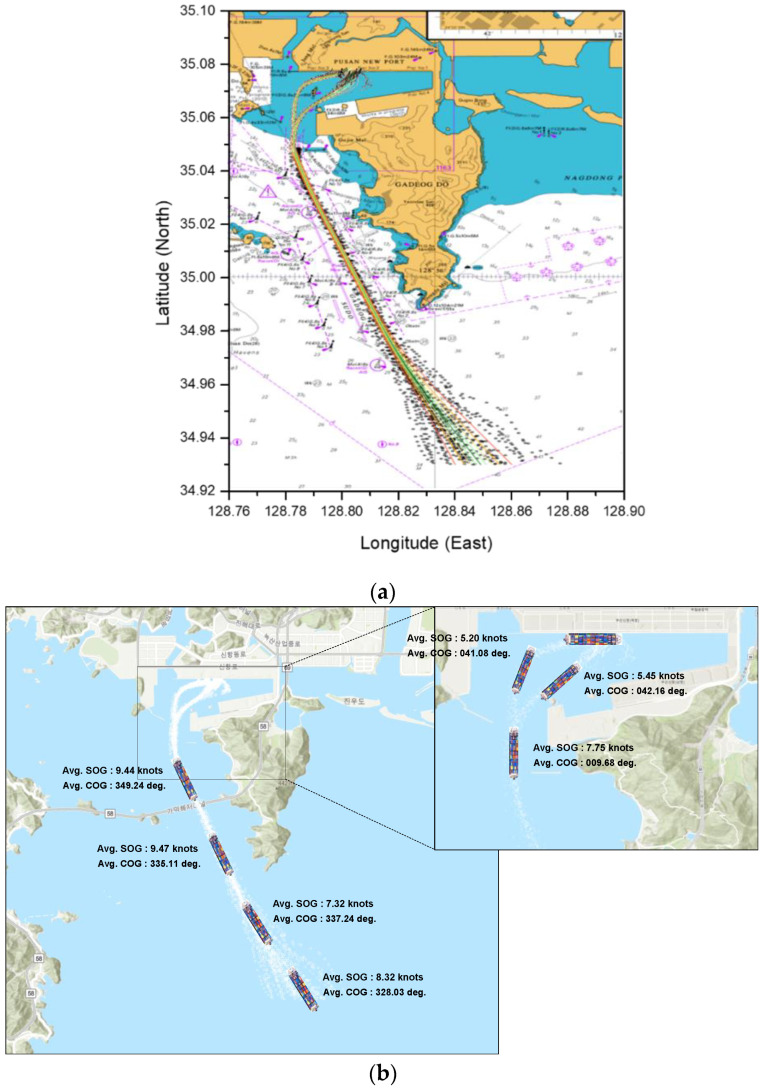
Example of using ship maneuvering guidelines in port: (**a**) ship’s position guidelines; (**b**) speed over ground and course over ground guidelines.

Because the quantile regression used in the study can effectively process data, including variability and uncertainty, it is suitable for analyzing the data of ships with various trajectories according to weather conditions and traffic flow. In addition, because regression by quantiles is possible, the operating range for ship maneuvering can be suggested, making the method effective for suggesting guidelines. Thus, the quantile regression model in this study proposes safe navigation guidelines, such as COG operation within the 10-quantile to 90-quantile range and SOG operation within the 40-quantile to 60-quantile range, based on the port situation. According to the analysis results, the performance of the QRNN model is better than that of GAMs, but both methodologies can be used. GAMs have the advantage of providing results using a smooth fitting line as a smoothing function, and the QRNN is complex but provides accurate analysis results based on datasets. Figure 9a shows an example of the use of guidelines for the ship’s position. In particular, it is possible to use these guidelines by plotting them on the Electronic Chart Display and Information System used in ships. In addition, linking the SOG and COG data, as shown in Figure 9b, will be helpful for the safe operation of ships by referring to the operating range at the relevant location.

The long-term utilization aspect of this study can be presented as a basic study related to the port operation of maritime autonomous surface ships (MASSs). Although many studies have dealt with autonomous navigation techniques in the ocean, relatively few have explored the autonomous navigation of ships in ports [16]. Therefore, various approaches based on data-based machine learning and artificial intelligence are required for autonomous ship operation in ports, and the results of this study can serve as basic data. Moreover, combining this study with research on the berthing of ships can provide the key to the connection technology between MASSs and ports [42,43].

## 5. Conclusions and Future Work

Quantile regression-based GAMs and a QRNN were applied for realizing guidelines for safe ship operation in ports using AIS-based ship trajectory data. The novelty of this work is that the SOG, COG, and ship’s position information can be analyzed by quantile regression to utilize the ship’s operating guidelines. Traditional statistical models based on mean values cannot interpret ship trajectory data with variability and uncertainty. A ship’s trajectory changes because of weather, traffic flow, and the pilot’s operation; however, the ships are safely berthed to complete the operation in the port. Therefore, proposing ship operation guidelines based on average values is subject to significant error. Due to the limitations of traditional statistical analysis, this study analyzed ship trajectory data via quantile regression analysis. This approach examines changes in SOG, COG, and other metrics with respect to the ship’s position via quantiles and can determine the ship’s maneuvering guideline range based on port conditions.

However, only the ship trajectory data of one port, i.e., Busan New Port, were used in this study. Hence, it is necessary to analyze various port and ship types, including a range ships’ sizes. Further, the performance of GAMs and the QRNN algorithm should be improved for safe maneuvering guidelines in the future. Data reliability should be further increased by collecting data for a long period of time, and additional research on the operator behavior and ship operation patterns according to the weather is necessary.

## Figures and Tables

**Table 1:** Characteristics of AIS data for analysis.

Categorization	AIS Information
Period	1 January 2020–30 April 2020
Collection Area	Latitude 034.8 N–035.1 N Longitude 128.7 E–129.0 E (Around Busan New Port)
Pier	Pier 2 No. 4 Pier 2 No. 5 Pier 3 No. 1
Ship Type	Container Ship
Size of ship	Gross tonnage 100–220k
Information	Ship’s position (latitude, longitude) Speed over Ground (knots) Course over Ground (degree)

**Table 2:** Number of times and the sides on which ships passed Todo.

Todo Passing	Pier			Total
	Pier 2 No. 4	Pier 2 No. 5	Pier 3 No. 1	
Left	5	17	1	23
Right	23	4	-	27

**Table 3:** Results of the minimum and maximum values of each parameter for normalization.

Phase		Speed over Ground (Knots)		Course Over Ground (Degree)		Longitude (East)		Latitude (North)	
		Min	Mas	Min	Max	Min	Max	Min	Max
Entering Phase		0.9	14.4	293.5	007.8 (367.8)	128.780	128.877	34.930	35.050
Berthing Phase	Left	0.0	12.0	184.5	179.8 (539.8)	128.781	128.805	35.050	35.078
	Right	0.0	10.2	191.3	173.7 (533.7)	128.781	128.811	35.050	35.078

**Table 4:** Descriptive statistics for ship trajectory data.

Group ^1^	SOG ^2^		COG ^3^		Longitude	
	Mean	Std.	Mean	Std.	Mean	Std.
34.930	9.07	2.28	329.03	10.40	128.85	0.01
34.935	8.45	2.44	328.95	10.23	128.85	0.01
34.940	8.29	2.53	330.16	11.00	128.84	0.01
34.945	8.44	2.13	330.19	11.70	128.84	0.01
34.950	8.30	1.92	329.10	10.90	128.83	0.01
34.955	8.24	1.94	328.82	10.83	128.83	0.01
34.960	8.11	2.19	330.97	8.15	128.83	0.01
34.965	8.11	1.78	332.45	7.59	128.82	0.01
34.970	7.76	1.49	334.94	9.22	128.82	0.01
34.975	7.69	1.65	334.95	6.53	128.82	0.01
34.980	7.78	1.92	336.18	4.86	128.81	0.01
34.985	7.79	1.88	338.68	7.14	128.81	0.01
34.990	8.42	2.08	338.17	3.23	128.81	0.01
34.995	9.27	2.36	338.42	2.60	128.81	0.01
35.000	9.80	1.85	338.19	2.35	128.80	0.01
35.005	10.53	1.79	338.59	1.68	128.80	0.01
35.010	10.73	1.67	338.54	1.76	128.80	0.01
35.015	10.97	1.42	337.68	1.57	128.80	0.01
35.020	10.87	1.48	336.63	2.16	128.79	0.01
35.025	10.66	1.34	336.27	2.47	128.79	0.01
35.030	10.60	1.31	335.80	2.77	128.79	0.01
35.035	10.43	1.43	337.06	2.76	128.79	0.01
35.040	10.06	1.28	343.79	5.73	128.78	0.01
35.045	9.38	1.36	353.08	4.86	128.78	0.01
35.050	8.62	1.26	359.07	3.30	128.78	0.01
35.055	8.16	1.16	363.07	4.79	128.78	0.01
35.060	7.48	1.15	014.97	12.56	128.78	0.01
35.065	6.65	1.19	032.51	21.38	128.79	0.01
35.070	4.77	1.86	042.87	18.27	128.79	0.01
35.075	1.17	1.20	031.33	46.24	128.80	0.01

^1^ Group units are latitude (North) ± 0.025, ^2^ speed over ground (knots), ^3^ course over ground (degree).

**Table 5:** Determination of the optimal number of hidden layers and the appropriate number of neurons in the hidden layer.

Hidden Layer	Neurons ^1^	MAE ^2^	Computation Time (s)
2	4	16.528711	10 s
	8	16.545627	10 s
	16	16.787603	11 s
	32	16.524037	15 s
3	4	16.499576	12 s
	8	16.391850	12 s
	16	16.671966	13 s
	32	16.369946	17 s
4	4	16.201253	14 s
	8	16.292288	15 s
	16	16.193952	15 s
	32	16.668355	17 s
5	4	16.218435	15 s
	8	16.382307	15 s
	16	16.237098	18 s
	32	16.383952	20 s

^1^ The number of neurons each hidden layer, ^2^ mean absolute error.

**Table 6:** The evaluation result of regression model according to quantile loss.

Model	Phase		Information	Quantile									Total
				0.1	0.2	0.3	0.4	0.5	0.6	0.7	0.8	0.9	
Quantile GAMs	Entering Phase		SOG	5.465	7.046	7.877	8.267	8.283	7.960	7.261	6.093	4.174	6.936
			COG	6.238	6.807	6.998	6.944	6.686	6.221	5.516	4.521	3.203	5.904
			LONG	14.398	15.429	16.162	16.752	17.249	17.666	17.997	18.220	18.262	16.904
	Berthing Phase	Left	SOG	20.124	19.827	18.600	16.870	14.796	12.425	9.786	6.874	3.692	13.666
			COG	2.568	3.386	4.118	4.796	5.426	6.012	6.547	7.010	7.353	5.246
			LONG	3.993	7.726	11.328	14.822	18.212	21.480	24.590	27.447	29.815	17.713
		Right	SOG	28.796	28.128	26.381	24.119	21.501	18.604	15.441	11.963	8.040	20.330
			COG	3.233	3.552	3.611	3.536	3.366	3.108	2.750	2.253	1.485	2.988
			LONG	6.310	9.582	12.368	15.529	18.308	20.974	23.502	25.841	27.799	17.801
QRNN	Entering Phase		SOG	4.503	6.336	7.467	8.026	8.196	7.932	7.221	5.463	3.247	6.488
			COG	5.785	6.797	7.077	6.945	6.359	5.747	4.898	3.831	2.305	5.527
			LONG	13.035	14.716	15.608	16.345	16.885	17.403	17.836	18.084	18.166	16.453
	Berthing Phase	Left	SOG	17.964	18.230	17.706	16.726	15.561	13.531	10.633	7.359	3.978	13.521
			COG	2.431	3.309	4.123	4.840	5.446	5.878	6.168	6.712	7.418	5.147
			LONG	4.224	8.098	11.491	14.836	18.253	21.472	24.216	26.734	28.745	17.563
		Right	SOG	27.056	26.949	25.644	23.889	21.625	18.536	15.409	11.951	7.963	19.891
			COG	2.179	2.815	3.001	2.990	3.093	2.846	2.438	1.935	1.166	2.496
			LONG	5.829	9.398	12.511	15.381	18.121	20.724	23.185	25.339	26.972	17.496

## Data Availability

Restriction apply to the availability of these data. Data were obtained from the Busan Port Authority.
